# Impact of DRG policy on the performance of tertiary hospital inpatient services in Chongqing, China: an interrupted time series study, 2020–2023

**DOI:** 10.3389/fpubh.2025.1523067

**Published:** 2025-03-05

**Authors:** Yunyu Liu, Zusong Liao, Juntao Tan, Yongjie Yan, Yuting Wang

**Affiliations:** ^1^Affiliated Banan Hospital of Chongqing Medical University, Chongqing, China; ^2^College of Medical Informatics, Chongqing Medical University, Chongqing, China; ^3^Department of Information, Daping Hospital, Army Medical University, Chongqing, China; ^4^Department of Public Health, Second Affiliated Hospital of Chongqing Medical University, Chongqing, China

**Keywords:** DRG, performance evaluation, service capacity, service efficiency, service cost interrupted time series

## Abstract

**Background:**

Implementing the diagnosis-related groups (DRG) payment policy in 2021 marked a significant step in increasing the capacity and efficiency of public hospital services in Chongqing, China. However, the adaptability and effectiveness of DRG policy in middle-income regions like Chongqing remain understudied. This study evaluates the impact of DRG on tertiary hospital inpatient services in Chongqing, focusing on challenges unique to resource-constrained settings.

**Methods:**

Using an interrupted time series design, we analyzed monthly data of 14 DRG performance measures obtained from the DRG comprehensive management system, covering two public hospitals in Chongqing from 2020 to 2023. To evaluate both immediate and long-term effects of the DRG policy, we employed an interrupted time series analysis model to analyze changes in indicator levels and trends pre- and post-intervention.

**Results:**

We found significant changes in the following indicators since the implementation of the DRG policy: case-mix index (CMI) level increased by 0.0661 (*p* = 0.02), but the trend decreased by 0.0071 (*p* < 0.001). The time efficiency index (TEI) level decreased by 0.123 (*p* < 0.001), while the trend increased by 0.0106 (*p* < 0.001). The cost efficiency index (CEI) level decreased by 0.0633 (*p* = 0.003), with the trend rising by 0.0076 (*p* < 0.001). And average length of stay (ALOS) trend increased by 0.0609 (*p* = 0.002). Readmission rates (RR) exhibited an instantaneous increase of 0.5653% (*p* = 0.008) post-intervention, though the long-term trend remained stable (*p* = 0.598). No significant differences were observed in the changes in inpatient numbers, surgical proportion, bed turnover rate (BTR), mortality rates (DR), cost per hospitalization (CPH), drug cost per hospitalization (DCPH), consumable cost per hospitalization (CCPH), medical examination cost per hospitalization (MECPH), or medical service cost per hospitalization (MSCPH).

**Conclusion:**

The DRG policy in Chongqing led to unintended trade-offs: tertiary hospitals prioritized high-volume, low-complexity cases, eroding service capacity for severe conditions. Middle-income regions faced implementation barriers, including fragmented health IT systems and insufficient administrative capacity, which diminished policy effectiveness. Policymakers must tailor DRG implementation to local contexts, balancing efficiency with equity and quality.

## 1 Introduction

With the growing demand for healthcare services and rising medical insurance expenditures, the healthcare systems in most countries face increasing pressure, particularly in China (1, 2). China has extensive social insurance coverage, and public hospitals, which are government-controlled, play a central role in healthcare provision (3). In the past, inpatient medical services in China primarily operated on a fee-for-service basis, often leading to overtreatment and limited improvement in service performance (4). Therefore, reforming medical insurance payments is crucial to alleviating the pressure on medical insurance expenditures. As a prepayment system, the Chinese government introduced the diagnosis-related groups (DRG) payment system, which combines capacity, efficiency, and quality of medical services (5).

Originating in the United States in the 1970s, many countries and regions have adopted the DRG payment method or inpatient care to contain expenditures and enhance transparency, efficiency, and quality in healthcare (6). This system categorizes similar clinical cases into groups based on patients’ diagnoses, age, sex, and complications, setting a predefined payment standard for each group. DRG have achieved positive results in the United States, where the growth rate of medical expenses and the length of stay were significantly reduced in New Jersey after applying the DRG payment system (7). However, in Germany, the DRG payment system significantly increased hospital activity by around 20% but did not necessarily shorten the average length of stay (8). The Chinese government first planned to introduce the DRG payment into medical insurance reform in Beijing in 2010 (9). Then, in 2015, Shanghai also introduced the DRG payment system to reward desired hospital performance and enhance inpatient service capacity (10). Findings from Both cities revealed that the DRG policy improved the capacity and efficiency of regional inpatient service. However, the classification rules of DRG require high–quality medical records, advanced information systems, and capable management teams typically found in economically developed regions (11). Consequently, implementing the DRG policy presents challenges in middle-income and low-income areas with limited resources (12).

Before 2020, no province in China had fully implemented the DRG payment system across all public hospitals, primarily due to limited IT infrastructure and underdeveloped information systems. As the conditions improved, the Chinese government announced the implementation of the DRG payment system in 30 pilot national monitoring cities in 2019 (13). Chongqing, one of the first cities to pilot DRG reform, included four medical institutions in December 2021 and implemented DRG payment based on the CHS-DRG (1.0 revised version) grouping scheme (14). Unlike Shanghai and Beijing, Chongqing’s healthcare system faces challenges such as urban–rural inequities and limited health IT maturity. Existing studies on DRG in China focus on affluent regions, overlooking contextual barriers in less-developed areas. Meanwhile, the hierarchical system mandates tertiary hospitals to manage severe cases and train lower-tier facilities. However, DRG payment reforms risk distorting this role by incentivizing hospitals to optimize revenue through high-volume, low-complexity cases (15). This study addresses this gap by evaluating DRG implementation in Chongqing’s tertiary hospitals, which serve as regional hubs for complex care under China’s hierarchical medical system. Meanwhile our study uniquely examines how DRG interact with hierarchical reforms, providing insights into systemic trade-offs between efficiency and equity.

## 2 Materials and methods

### 2.1 Data sources

Chongqing City, the largest of China’s directly administered municipalities, is located in the Southwest of China and covers an area of 82,402 km^2^. In 2019, Chongqing had a population of 31.24 million. The per capita GDP in Chongqing was $10,605 (2019), which did not exceed the high-income country threshold of ($12,235), but positioned Chongqing as a leader in Southwest China (16). Therefore, the experience of implementing the DRG policy in Chongqing was crucial for the comprehensive implementation of this policy in southwest China. Chongqing made upfront preparations for the DRG payment policy starting October 24, 2019. In 2020, the Chongqing Healthcare Security Administration established a DRG comprehensive management system and collected base data from the past 3 years from 82 medical institutions in the city that offer hospitalization services to conduct disease grouping and cost measurement. After grouping by DRG, hospitalization cases were divided into 778 DRG groups, of which 465 DRG were internal medicine groups, 32 were non-operating room treatment groups, and 295 were surgical groups. On November 26, 2021, the DRG Payment Implementation Rules were issued, and four tertiary hospitals were selected as pilot sites, with implementation scheduled for December 2021. This study’s data were obtained from two of the four pilot hospitals in Chongqing, encompassing 486,576 discharged and enrolled patients from 2020 to 2023. The platform has been verified the quality of the data uploaded by each hospital by logic verification and key quality control index monthly.

### 2.2 Research indicators

The data include four dimensions: medical service capacity, medical service efficiency, medical service quality and medical expenses. The metrics for assessing medical service capacity include the number of inpatient cases, the Case Mix Index (CMI), and the surgery rate among the inpatient cases (17). A higher CMI indicates greater complexity and severity of the diseases being treated. CMI is a measure used in healthcare to assess the complexity and resource intensity of a group of patients treated in a hospital or healthcare facility. Surgery rate refers to the proportion of surgical procedures performed among all admissions or patients treated within a hospital or healthcare facility during a specific period. Medical service efficiency refers to the effectiveness and productivity of healthcare services provided within a medical facility or system. It encompasses four indicators: Average length of stay (ALOS), time efficiency index (TEI), cost efficiency index (CEI), and bed turnover rate (BTR). ALOS refers to the average duration of time that patients spend in a hospital or healthcare facility during a single admission. CEI and TEI measure the ratio of actual costs or length of stay to regional standards. A value near 1 aligns with regional averages. Values <1 indicate lower costs or shorter stays, while values >1 reflect higher costs or prolonged stays. Medical service quality was evaluated using mortality rates (DR), defined as the proportion of deaths among hospitalized patients, and readmission rates (RR), calculated as the percentage of patients readmitted within 30 days of discharge (18, 19). The cost evaluation indicators used in this study were: cost per hospitalization (CPH), drug cost per hospitalization (DCPH), consumable cost per hospitalization (CCPH), medical examination cost per hospitalization (MECPH), Medical service cost per hospitalization (MSCPH), These indicators are commonly used in relevant studies (20–22).

### 2.3 Research methods

Chongqing began implementing the DRG payment reform policy in December 2021. This study takes December 2021 as the reference time point, dividing the research into two periods: before the DRG payment reform (January 2020–November 2021) and after the DRG payment reform (December 2021–December 2023). The data were sorted and statistically analyzed using Excel, with descriptive statistics conducted on an annual basis. This study employs the Interrupted Time Series Analysis (ITSA) model, which compares changes before and after an intervention to better understand the immediate and long-term changes following policy implementation (23). ITSA is a quasi-experimental design method used to evaluate an intervention’s effectiveness retrospectively. It necessitates measuring indicators at multiple time points before and after the intervention and analyzing observed changes to determine whether they are due to long-term trends or attributable to the intervention. This approach allows for the evaluation of the outcome of level changes and trends before and after the intervention implementation (24, 25). By employing the ITSA segmented regression method, a multivariate regression equation was constructed to conduct a comparative analysis of the changes in medical service difficulty, efficiency, and cost indicators before and after the implementation of the policy. This approach focused on verifying their fluctuation trends and evaluating the policy’s impact on these indicators. The general formula for the ITSA time series model was as follows (25, 26):


$$Y_{t}=\beta_{0}+\beta_{1}\times X_{\mathrm{time}}+\beta_{2}\times X_{int\mathrm{ervention}}+\beta_{3}\times X_{\mathrm{posttime}}+e_{t}$$

In this context, Y_t_ represents the dependent variable, which denotes the observed indicator value at time t. X_time_ is a continuous time series that signifies the frequency of time units since the commencement of the study, with X_time_ = 1, 2, 3, n. This research is conducted monthly, and observation points are sequentially assigned values from “0” to “47.” X_intervention_ is a binary categorical variable, assigned a value of “0” before the intervention (X_intervention_=0) and “1” after the intervention (X_intervention_=1). X_posttime_ represents the time series after the intervention, with pre-intervention observation points assigned the value of “0” and post-intervention points assigned values from “1 to 24.” e_t_ represents the error term. β_0_ the baseline level is at *t* = 0, a constant term. The x represents the trend change before the implementation of the DRG policy in this study. β_1_ indicates the pre-intervention trend change Y_t_, representing the slope before the implementation of the DRG policy. β_2_ represents the instantaneous level change before and after the implementation of intervention measures. In this context, it denotes the magnitude of change Y_t_ caused by the implementation of the DRG policy in the first month following its enforcement. β_3_ represents the change in trend after the implementation of the intervention, indicating the change in slope of Y_t_ following the DRG policy. β_1_ + β_3_ represents the post-intervention slope, indicating the altered trend following the implementation of DRG. To account for COVID-19’s confounding effects, we conducted sensitivity analyses by excluding data from pandemic peak periods (January–April 2020, August–October 2021, November 2022–January 2023). These intervals corresponded to localized lockdowns and reduced hospital admissions in Chongqing. We re-ran the interrupted time series (ITS) model using the truncated dataset to assess whether the observed DRG policy effects persisted after adjusting for pandemic-related disruptions.

### 2.4 Statistical analysis methods

We used Python software (version 3.10) to construct an interrupted time series analysis model for statistical analysis and data visualization, considering a significance level of *p* < 0.05 as indicative of statistical significance. Model validation included residual analysis and Q-Q plots to assess normality and homoscedasticity. The goodness-of-fit was evaluated using R^2^ values, with higher values indicating robust model performance.

## 3 Results

### 3.1 Basic overview

From 2020 to 2023, there was a significant increase in the number of inpatients at two tertiary hospitals in Chongqing. The CMI value exhibited an increasing trend from 2020 to 2022, followed by a decreasing trend in 2023. TEI, CEI, and ALOS decreased yearly, with ALOS displaying a particularly pronounced downward trend in 2022—the proportion of surgeries declined by the year, with the most rapid decrease occurring in 2021. DR fluctuated minimally (0.87% in 2020 to 0.66% in 2023), while RR increased from 1.86 to 2.29% during the same period. The bed turnover rate revealed an upward trend annually, with the fastest increase in 2023, and regarding cost indicators, CPH, DCPH, MECPH, and MSCPH gradually decreased yearly. However, CCPH indicates a slight rise in 2022 but decreased significantly in 2023. Detailed data can be found in Table 1.

**Table 1:** Medical performance indicators of two tertiary hospitals between 2020 and 2023.

Indicator	2020	2021	2022	2023
Inpatients number	150,602	174,393	179,443	206,306
CMI	1.1508	1.2181	1.2832	1.2305
Surgical proportion (%)	56.57	49.17	44.32	42.63
TEI	1.38	1.2	1	0.97
CEI	1.32	1.19	1.05	0.99
ALOS	8.99	8.07	7.07	6.7
BTR	29.86	36.70	38.13	46.03
DR (%)	0.87	0.67	0.73	0.66
RR (%)	1.86	1.71	2.40	2.29
CPH	2,886.71	2,715.51	2,602.88	2,357.29
DCPH	738.32	665.52	625.3	525.2
CCPH	695.47	654.23	662.71	605.35
MECPH	757.79	723.05	651.11	572.35
MSCPH	600.2	581.33	560.24	479.94

### 3.2 Impact of DRG policy implementation on the medical service capacity of public hospitals

**Figure 1:**
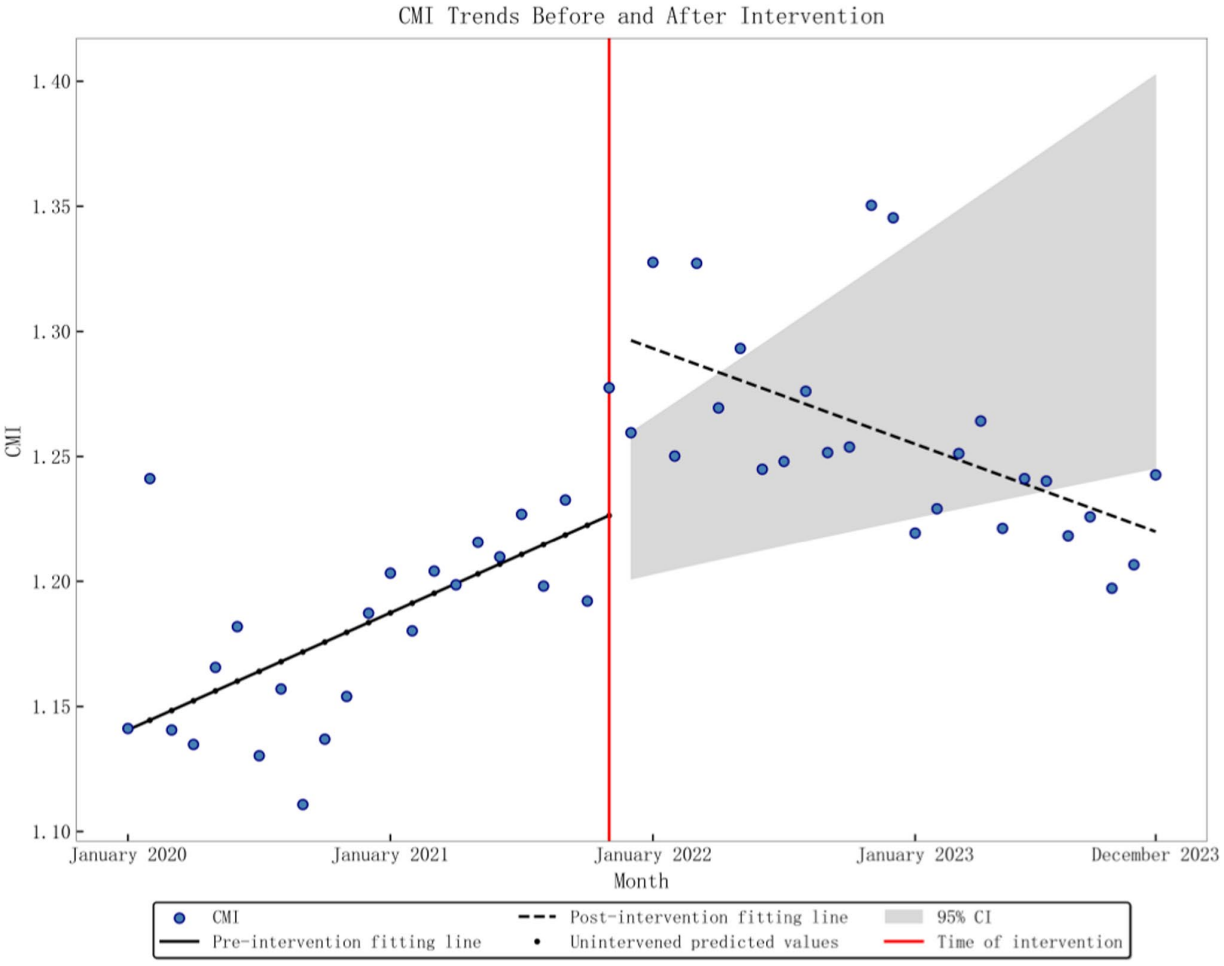
Graphic of CMI change in performance indicators pre- and post-policy intervention.

The baseline monthly average number of hospitalized patients at two tertiary hospitals was 11,337.33. Before the DRG policy, there was a significant upward trend (*p* = 0.001), with an increase of 192 monthly patients. However, after implementation, neither the immediate level nor the long-term trend reveals significant changes (*p* = 0.284, *p* = 0.264). The average monthly baseline CMI level was 1.1406, with a significant upward trend before implementing the DRG policy (*p* = 0.001), increasing to 0.039 per month. After implementation, the instantaneous level rose by 0.0661 (*p* = 0.002), and there was a clear downward trend (*p* < 0.001), with the slope decreasing by 0.0071 compared to pre-implementation, as depicted in Figure 1. The baseline average monthly surgical ratio was 61.96%. Before the DRG policy, there was a significant downward trend (*p* < 0.001), with a monthly decrease of 0.45%. After implementation, there was an instantaneous decrease of 5.88% (*p* = 0.017) but a non-significant change in the long-term trend (*p* = 0.164). Detailed data can be found in Table 2.

**Table 2:** Results of change in performance indicators pre- and post-policy intervention.

Indicator	R^2^	β0		β1		β2		β3	
		Value	*p*	Value	*p*	Value	*p*	Value	*p*
Inpatients number	0.499	11,109	<0.001	194.72	0.001	−991.96	0.284	79.35	0.264
CMI	0.654	1.1406	<0.001	0.0039	0.001	0.0661	0.002	−0.0071	<0.001
Surgical proportion (%)	0.776	62.4087	<0.001	−0.4488	0.001	−6.1291	0.012	0.2416	0.164
TEI	0.942	1.4474	<0.001	−0.0132	<0.001	−0.123	<0.001	0.0106	<0.001
CEI	0.944	1.3965	<0.001	−0.0115	<0.001	−0.0633	0.003	0.0076	<0.001
ALOS	0.836	9.62	<0.001	−0.0874	<0.001	−0.3676	0.163	0.0609	0.002
BTR	0.506	2.1238	<0.001	0.06	0.001	−0.3343	0.261	−0.0229	0.286
DR (%)	0.067	1.1883	<0.001	−0.0313	0.095	0.3052	0.372	0.0298	0.229
RR (%)	0.394	1.8380	<0.001	−0.0051	0.642	0.5653	0.008	0.0078	0.598
CPH	0.740	3,091.57	<0.001	−20.68	<0.001	87.43	0.272	3.98	0.489
DCPH	0.587	842.18	<0.001	−9.87	<0.001	43.98	0.325	4.26	0.192
CCPH	0.496	735.55	<0.001	−4.63	0.001	63.13	0.011	0.11	0.951
MECPH	0.821	779.31	<0.001	−2.66	0.005	−51.35	0.004	0.16	0.895
MSCPH	0.587	632.31	<0.001	−2.92	0.006	16.53	0.379	−0.42	0.755

### 3.3 Impact of DRG policy implementation on the medical service efficiency of public hospitals

**Figure 2:**
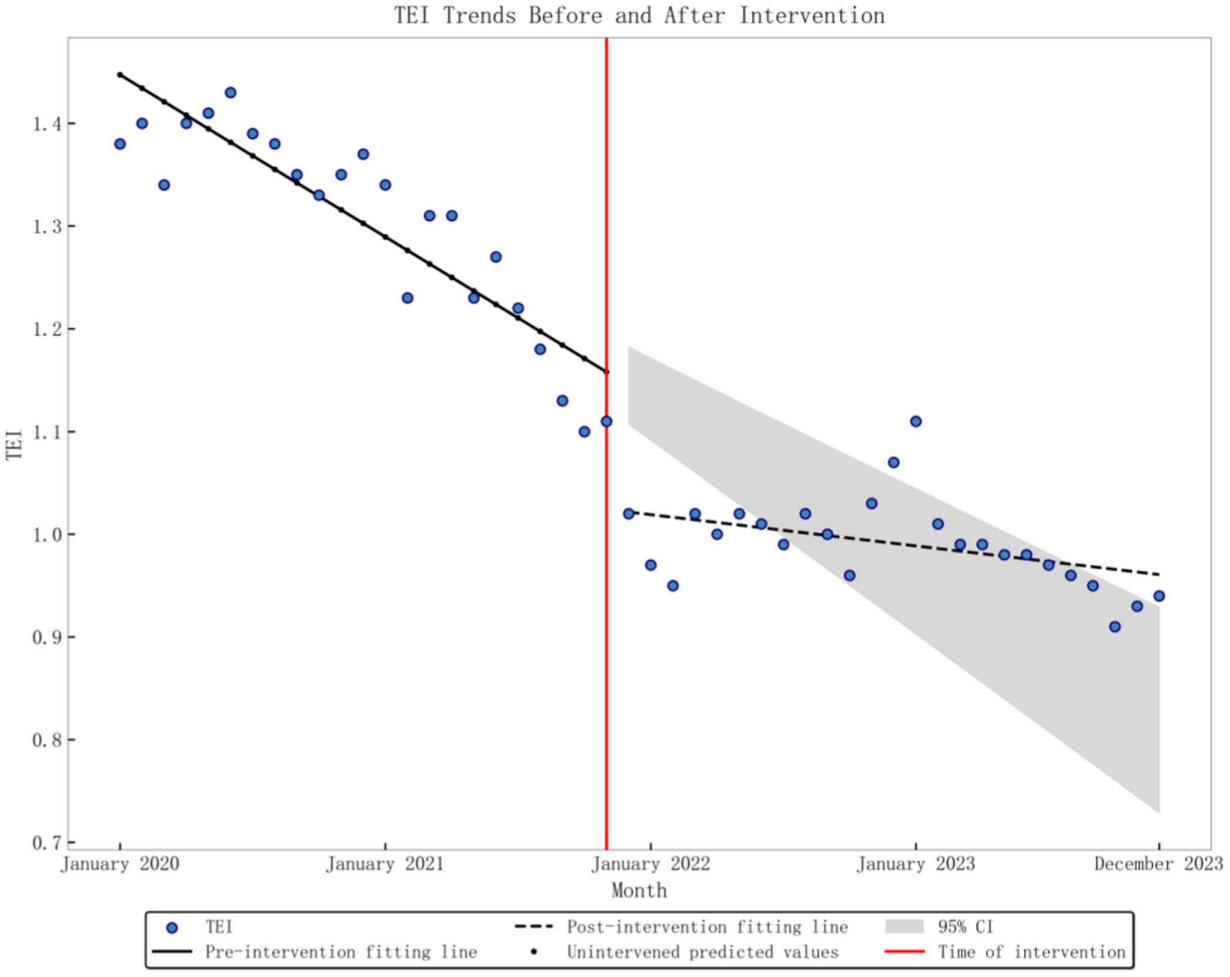
Graphic of TEI change in performance indicators pre- and post-policy intervention.

**Figure 3:**
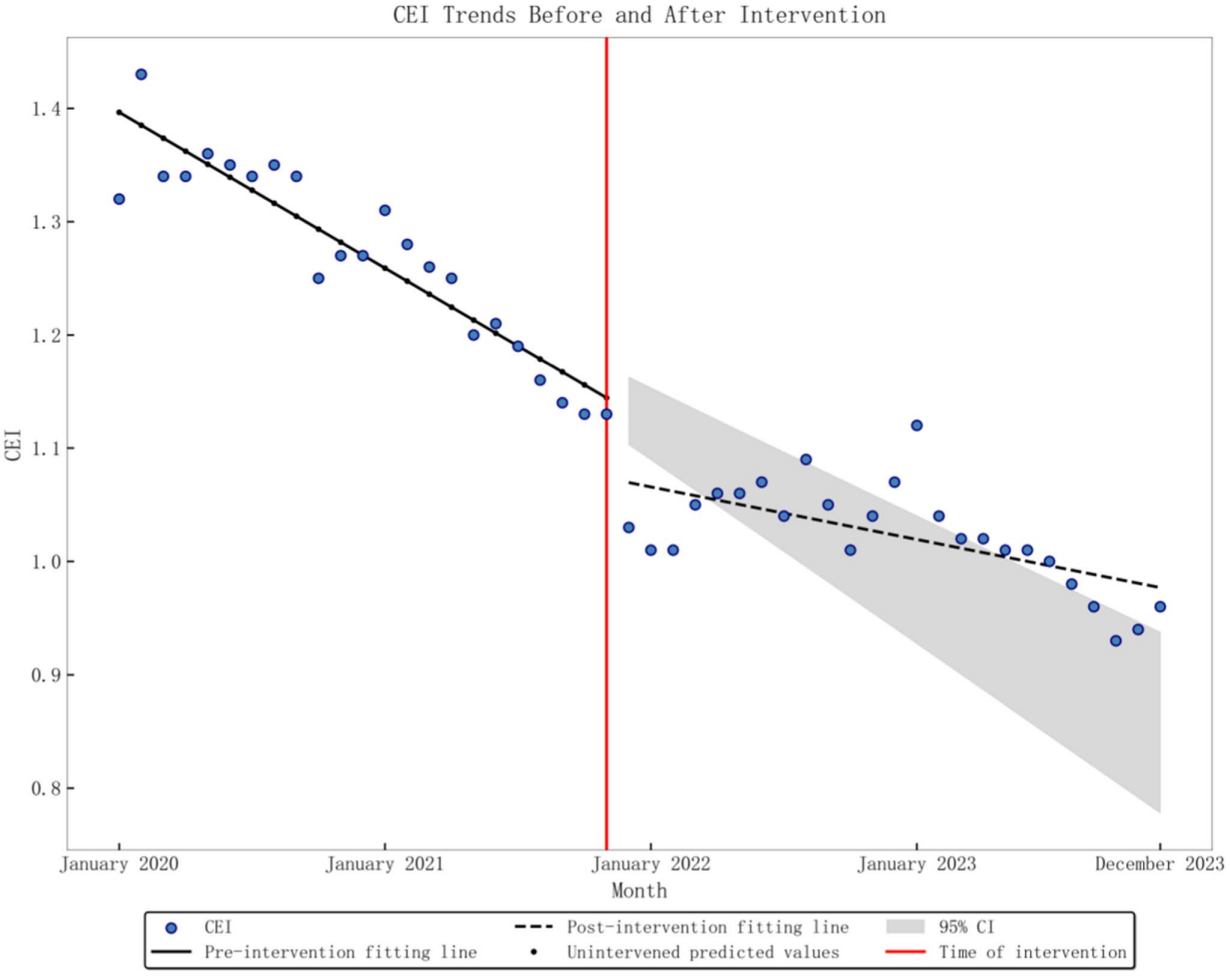
Graphic of CEI change in performance indicators pre- and post-policy intervention.

**Figure 4:**
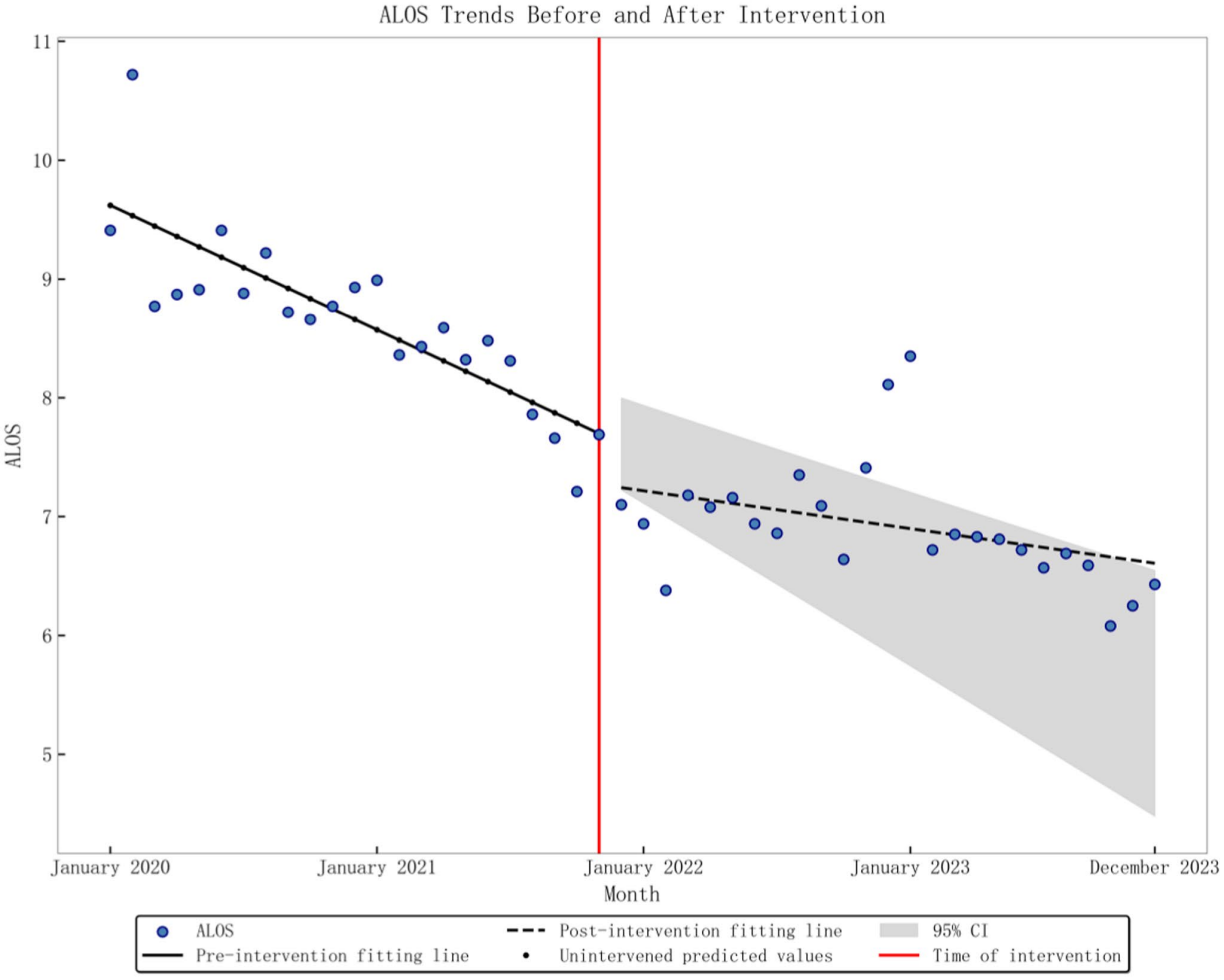
Graphic of ALOS change in performance indicators pre- and post-policy intervention.

The baseline level of the average monthly TEI was 1.4474, and there was a significant downward trend (*p* < 0.001) before the DRG policy implementation, decreasing by 0.0132 per month. After implementation, there was an instantaneous decrease of 0.123 (*p* < 0.001), and the slope increased by 0.0106 compared to pre-implementation, but overall, the trend remained downward (*p* < 0.001), as depicted in Figure 2. The baseline level of monthly CEI was 1.3965, with a significant downward trend (*p* < 0.001) observed before the implementation of the DRG policy, decreasing by 0.0115 per month. After implementation, there was an instantaneous decrease of 0.0633 (*p* = 0.003), and the slope increased by 0.0076 compared to pre-implementation levels. However, the overall trend remained downward (*p* < 0.001), as depicted in Figure 3. The average monthly length of hospital stay was 9.62 days at baseline, with a significant downward trend before the DRG policy implementation (*p* < 0.001), decreasing by 0.0874 days per month. After implementation, there was no substantial change in the instantaneous level (*p* = 0.163), but the slope increased by 0.0609 compared to pre-implementation. However, the overall trend remained downward (*p* = 0.002), as found in Figure 4. The baseline average monthly BTR was 2.1238, with a significant upward trend before the DRG policy implementation (*p* = 0.001), increasing by 0.06 per month. After implementation, there were no substantial changes in the instantaneous level or long-term trend (*p* = 0.261; *p* = 0.286). Detailed data can be found in Table 2.

### 3.4 Impact of DRG policy implementation on the medical service quality of public hospitals

The baseline monthly mortality rate (DR) was 1.1883%, with no significant pre-intervention trend (*p* = 0.095). Post-implementation, neither immediate level changes (*p* = 0.372) nor long-term trends (*p* = 0.229) were observed. For readmission rates (RR), the baseline level was 1.8380%, showing no pre-intervention trend (*p* = 0.642). After the DRG policy, there was an instantaneous increase of 0.5653% (*p* = 0.008), but the long-term trend remained non-significant (*p* = 0.598; Table 2).

### 3.5 Impact of DRG policy implementation on the average per cost of inpatients in public hospitals

The baseline of CPH was $3,070.22, indicating a significant downward trend before the implementation of the DRG policy (*p* < 0.001), with a monthly decrease of $20.69. After implementation, there were non-significant changes in either the immediate level or long-term trend (*p* = 0.254, *p* = 0.489). The baseline DCPH was $832.31, and there was a significant downward trend before the implementation of the DRG policy (*p* < 0.001), with a monthly decrease of $9.87. Post implementation, there were non-significant changes in the immediate or long-term trend (*p* = 0.283, *p* = 0.192). The baseline level of CCPH was $730.93, with a significant downward trend before the DRG policy (*p* = 0.001), decreasing by $4.63. After the implementation, non-significant changes were observed in the long-term trend (*p* = 0.951). The baseline MECPH was $776.66, indicating a significant downward trend before the DRG policy implementation (*p* = 0.005), with a monthly decrease of $2.66. Following the policy implementation, non-significant long-term trend change was observed (*p* = 0.895). The baseline MSCPH was $629.38, and there was a significant downward trend before the DRG policy implementation (*p* = 0.006), with a monthly decrease of $2.92. Post implementation, there were non-significant changes in the immediate or long-term trend (*p* = 0.393, *p* = 0.755). Detailed data can be found in Table 2.

### 3.6 Model validation and residual analysis

To validate the normality assumption of the interrupted time series model, we generated Q-Q plots of residuals (Supplementary Figures S1–S4). The results indicate that the residuals for key indicators (CMI, TEI, CEI, ALOS) closely followed the theoretical quantile lines (Supplementary Figures S1–S4), suggesting that the residuals largely adhered to the normality assumption. For example, in the Q-Q plot for CMI (Supplementary Figure S1), most data points aligned closely with the diagonal line, with minor deviations observed in the higher quantiles, which may reflect extreme cases or unobserved confounders. Similarly, Q-Q plots for TEI and CEI (Supplementary Figures S2, S3) displayed symmetric residual distributions, supporting the validity of the model. The Q-Q plot for ALOS (Supplementary Figure S4) showed slight tail deviations, potentially attributable to asymmetric factors influencing hospital stays (e.g., rare prolonged stays for complex cases). Despite localized deviations, the overall model goodness-of-fit (R^2^ values in Tables 2, 3) and residual autocorrelation tests (Durbin-Watson statistics showed no significant autocorrelation) confirmed that the model robustly captured trend changes following the DRG policy intervention.

**Table 3:** Sensitivity analysis exclude pandemic peaks.

Indicator	R^2^	β0		β1		β2		β3	
		Value	*p*	Value	*p*	Value	*p*	Value	*p*
Inpatients number	0.419	12,560	<0.001	165.64	0.069	−636.60	0.540	−50.26	0.616
CMI	0.689	1.1248	<0.001	0.0069	<0.001	0.0639	0.003	−0.0101	<0.001
Surgical proportion (%)	0.845	57.8127	<0.001	−0.1481	0.365	−9.4858	<0.001	−0.0591	0.746
TEI	0.949	1.4477	<0.001	−0.0157	<0.001	−0.1725	<0.001	0.0132	<0.001
CEI	0.943	1.3827	<0.001	−0.0130	<0.001	−0.1018	<0.001	0.0091	<0.001
ALOS	0.839	9.2710	<0.001	−0.0711	<0.001	−0.8640	<0.001	0.0446	<0.001
BTR	0.425	2.5077	<0.001	0.0453	0.078	−0.1716	0.558	−0.0114	0.686
DR (%)	0.001	0.7498	<0.001	−0.0012	0.970	0.0452	0.903	−0.0002	0.995
RR (%)	0.372	2.0093	<0.001	−0.0205	0.262	0.6023	0.007	0.0232	0.259
CPH	0.766	2,951.47	<0.001	−13.21	0.043	−36.89	0.618	−3.49	0.625
DCPH	0.629	750.38	<0.001	−4.88	0.115	−13.08	0.712	−0.73	0.831
CCPH	0.429	719.41	<0.001	−4.99	0.043	52.77	0.064	0.47	0.860
MECPH	0.885	775.24	<0.001	−1.79	0.177	−79.81	<0.001	−0.7103	0.629
MSCPH	0.642	611.99	<0.001	−1.39	0.369	−8.09	0.652	−1.95	0.262

### 3.7 Sensitivity analysis exclude pandemic peaks

Sensitivity analysis excluding pandemic peaks (Table 3) revealed comparable trends: CMI declined post-policy (1.2305 in 2023 vs. 1.2832 in 2022), surgical proportions dropped sharply (−9.49%, *p* < 0.001), and MECPH decreased significantly (−79.81, *p* < 0.001). Readmission rates (RR) remained elevated (2.29% in 2023), aligning with primary findings (Table 1). These adjustments reinforced the stability of DRG-related trends despite pandemic pressures.

## 4 Discussion

The ITSA results indicated that the CMI of two tertiary hospitals revealed a decreasing trend after implementing the DRG policy. These suggest that the DRG policy challenges enhancing medical service capacity for public hospitals. According to the CMI calculation formula, its value is related to the total weight and total number of cases admitted to the hospital. Table 1 indicates that the number of patients admitted between 2022 and 2023 significantly increased compared with patients from 2020 to 2021, alongside a decreasing trend in the surgical proportion. The CHS-DRG (1.0 revised version) categorizes DRG groups into three categories: internal medicine group, non-operating room treatment, and surgical. The average weight for the internal medicine group is 0.7730, for the non-operating room treatment group is 2.2700, and for the surgery group is 2.6057 (14). Therefore, it can be inferred that the decrease in CMI may be attributed to a reduction in the proportion of surgical patients within the hospital’s surgical department. Tingting Zhu’s study also found that the CMI mean in tertiary institutions changed later and improved slower than in secondary institutions after the change. Reasons for this phenomenon may include the increased number of low-related weight cases treated at tertiary institutions, which constrained the improvement in CMI (20). The decline in CMI and surgical proportions (*p* < 0.001) indicates that tertiary hospitals prioritized high-volume, low-complexity cases to maximize revenue under fixed DRG payments. While this strategy may sustain short-term income, it risks eroding hospitals’ capacity to manage severe diseases—a critical function of tertiary institutions (15). Over time, such shifts could strain regional healthcare systems if secondary hospitals are unable to absorb increased referrals for complex cases. Additionally, centralized procurement policies may offset DRG-induced cost pressures, but prolonged reliance on these measures could stifle innovation in medical technologies (27).

Our study found that, after the DRG policy intervention, the rate of decrease in TEI and CEI in two tertiary hospitals slowed as they approached a value of 1. In contrast, Lvfan Feng’s study indicated that TEI decreased more rapidly toward 1, and CEI shifted from upward to downward (10). These findings suggest that the DRG policy not only helped control the increases but also contributed to reducing ALOS. According to the formula for TEI and CEI, these values are influenced not only by the average level of medical institutions but also by the average level of the region (20). Although our study indicated that ALOS and CPH revealed a decreasing trend from 2020 to 2023 in the two tertiary hospitals, the ratio of the average level of these indicators from the region might have decreased more rapidly than the ratio for the hospitals themselves. This discrepancy could account for the slower reduction rate of TEI and CEI.

Furthermore, our study found that the decrease in ALOS decelerated following the DRG policy intervention. There were minimal changes in BTR and surgical proportions, with differences lacking statistical significance. However, Table 1 indicates that BTR has increased yearly while surgical proportions have decreased. Robert Messerle’s study also reported a decreased ALOS, though less pronounced than before the DRG system’s introduction (8). The study suggested that German hospitals with high (idle) capacities did not experience a significant reduction in overall length of stay due to the reform. A study in Spain on prolonged hospital stays found that internal medicine patients significantly contributed to the total stays and significantly during hospitalization (28). Based on our study’s findings, we infer that the increasing proportion of internal medicine patients, combined with high (idle) capacities, may have slowed the rate of decrease in ALOS. Consequently, our study suggests that the DRG policy has had a slight negative impact on medical service efficiency in these two tertiary hospitals.

The slowed reduction in ALOS (*p* = 0.002) and CEI/TEI trends may reflect strategic hospital adaptations. For example, internal medicine departments might prolong stays to avoid readmission penalties, whereas surgical departments could limit elective procedures to mitigate financial risks under DRG payment ceilings (29). These operational adjustments highlight the tension between efficiency targets and clinical autonomy—a challenge also noted in Taiwan’s DRG implementation (30). Meanwhile, although our data lack granular patient demographics, the rising BTR and declining surgical rates (*p* = 0.017) suggest potential disparities in access to specialized care. Vulnerable populations requiring complex surgeries (e.g., older adult patients with comorbidities) may face longer wait times or referral barriers if hospitals deprioritize high-weight DRG groups. Policymakers should mandate equity audits to monitor access gaps, particularly in regions with mixed urban–rural populations like Chongqing. Finally, Chongqing’s experience contrasts with high-income regions like Shanghai, where DRG policies accelerated efficiency gains (10). This disparity may stem from differences in health IT infrastructure and administrative readiness. In Germany, DRG adoption increased hospital activity without shortening ALOS, whereas American achieved both cost containment and efficiency (7, 8). These comparisons emphasize that DRG success depends on local health system maturity, necessitating phased implementation in resource-constrained settings.

Our analysis revealed no significant changes in DR following DRG implementation, aligning with studies in Beijing and Shanghai where DRG reforms did not compromise care quality. However, the transient spike in readmission rates (RR) post-intervention (*p* = 0.008) suggests potential unintended consequences, such as premature discharges to meet efficiency targets (10, 31). Similar trends were observed in Brazil, where DRG adoption shortened ALOS, however the hospital readmission rate increased (32). The stability of DR may reflect maintained clinical standards despite financial pressures, whereas rising RR underscores the need for post-discharge care coordination. These findings highlight the importance of balancing efficiency gains with patient-centered outcomes. Future reforms should integrate safeguards, such as enhanced follow-up protocols, to mitigate readmission risks.

Our study indicated that CPH, DCPH, CCPH, MECPH, and MSCPH exhibited a decreasing trend before and after the DRG policy implementation, with no significant differences in changes observed after the policy was implemented. A study conducted in Taipei, China, found that introducing DRG payment decreased the cost of medical services without a significant difference (30). The increasingly improved healthcare regulatory system and the implementation of centralized procurement of drugs and consumables in China since 2019 have significantly reduced CPH, DCPH, and CCPH (21, 22, 33). Based on our study’s results, we infer that the DRG policy has had minimal impact on inpatient expenses following the comprehensive deepening of medical insurance reform and the implementation of centralized procurement.

Sensitivity analyses excluding pandemic peaks (Table 3) confirmed the robustness of DRG-related trends, demonstrating that observed effects were not artifacts of COVID-19 disruptions. Post-policy declines in CMI (−0.0101/month) and surgical proportions (−9.49%) persisted, suggesting DRG-induced shifts toward low-complexity cases were not artifacts of pandemic disruptions. Similarly, the transient RR increase (0.6023%, *p* = 0.007) remained significant, aligning with global evidence of DRG-driven premature discharges (32). Notably, MECPH reductions (−79.81, *p* < 0.001) intensified in the pandemic-adjusted model, likely reflecting stricter cost controls under DRG. While pandemic pressures temporarily reduced elective surgeries and altered patient flows (34), our adjusted analysis demonstrated that DRG policy effects dominated long-term trends. For example, pre-pandemic declines in ALOS (−0.0711/month) accelerated post-DRG (−0.8640, *p* < 0.001), contradicting claims that pandemic-related bed shortages drove efficiency gains. These findings align with studies in Shanghai (10), where DRG reforms achieved sustained efficiency improvements despite external shocks.

In summary, to address implementation challenges, we recommend: (1) phased DRG adoption, prioritizing hospitals with advanced IT systems. (2) Targeted training programs for clinical coding and cost management. (3) Equity audits to monitor access disparities, particularly for rural populations.

This study has some limitations. First, our study focused on two tertiary hospitals in Chongqing due to data accessibility and their role as early DRG policy pilots. However, this limited sample may introduce selection bias, as pilot hospitals often receive additional administrative support compared to non-pilot institutions. Second, due to the limited period of the data, we used monthly rather than annual data to ensure enough data points. However, using the month as the unit may affect the interpretation of the level changes, as the influence of different month-on-level changes is unavoidable. Third, our findings are derived from two tertiary hospitals in Chongqing, a middle-income region. This focus limits generalizability to secondary hospitals or rural institutions, which may lack the advanced information systems and management capacity required for effective DRG implementation. For example, Zhu’s study reported that secondary hospitals in Wenzhou exhibited faster CMI improvements post-DRG (20), likely due to their lower baseline complexity. Future studies should compare DRG impacts across hospital tiers and regions to identify context-specific barriers. Finally, the impact of COVID-19 was ongoing in Chongqing from 2020 to 2022. The pandemic necessitated strict prevention and control measures whenever outbreaks occurred, which likely resulted in decreased patient visits and hospital revenues while increasing operation costs and affecting medical efficiency. These factors may have hindered the improvement of the selected indicators.

## 5 Conclusion

Our study identified significant variations in key performance indicators—such as case-mix index (CMI), time efficiency index (TEI), and readmission rates (RR)—before and after the implementation of the DRG policy in Chongqing. While the policy aimed to enhance medical capacity and standardize treatment costs across institutions, our findings indicate unintended consequences: tertiary hospitals experienced declines in both service capacity and operational efficiency. Notably, the rise in readmission rates (from 1.86 to 2.29%) underscores systemic gaps in care continuity, potentially linked to premature discharges driven by efficiency incentives. Meanwhile, Sensitivity analyses excluding COVID-19 peak periods affirmed the robustness of our findings: DRG implementation in Chongqing reduced service capacity and incentivized cost-cutting strategies. Hospital responses to DRG payment reforms were context-dependent, influenced by factors such as regional resource allocation and institutional management practices. These findings challenge the assumption that DRG policies universally improve healthcare outcomes and suggest that one-size-fits-all approaches may neglect local health system nuances. Policymakers should adopt incremental DRG implementation, starting with well-resourced hospitals, and provide subsidies for complex cases to prevent service erosion. In resource-constrained regions, hybrid payment models (e.g., DRG with capitation) may balance efficiency and equity.

## Data Availability

The data analyzed in this study is subject to the following licenses/restrictions: the data can be made public only upon the consent of the corresponding author. Requests to access these datasets should be directed to 303914@hospital.cqmu.edu.cn.
