# Supplementary figures and images for: Impact of DRG policy on the performance of tertiary hospital inpatient services in Chongqing, China: an interrupted time series study, 2020–2023
Source: Front Public Health. 2025 Mar 5;13:1523067. doi: 10.3389/fpubh.2025.1523067 (PMC11922081; doi:10.3389/fpubh.2025.1523067)

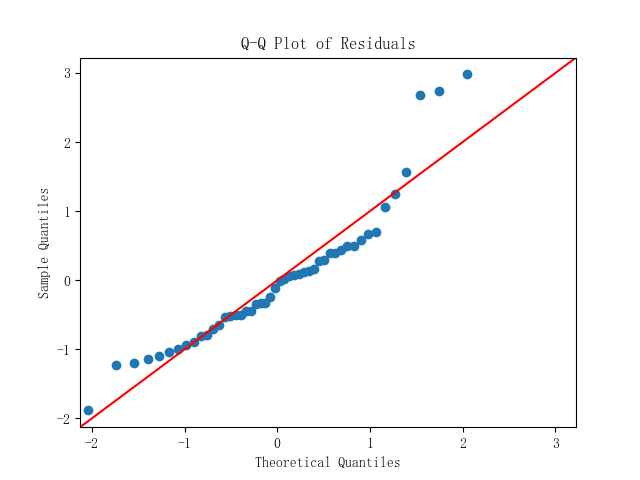

Supplement: Supplementary file 1 [file Image_1.png]

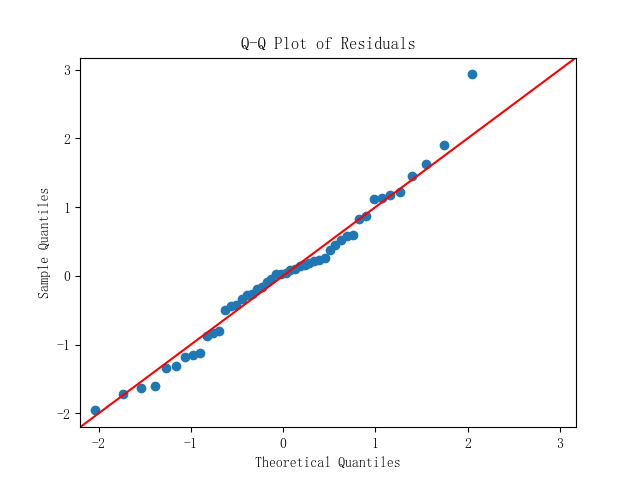

Supplement: Supplementary file 2 [file Image_2.png]

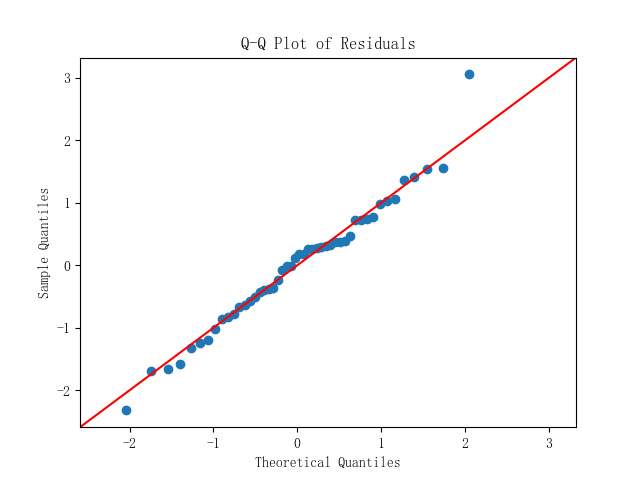

Supplement: Supplementary file 3 [file Image_3.png]

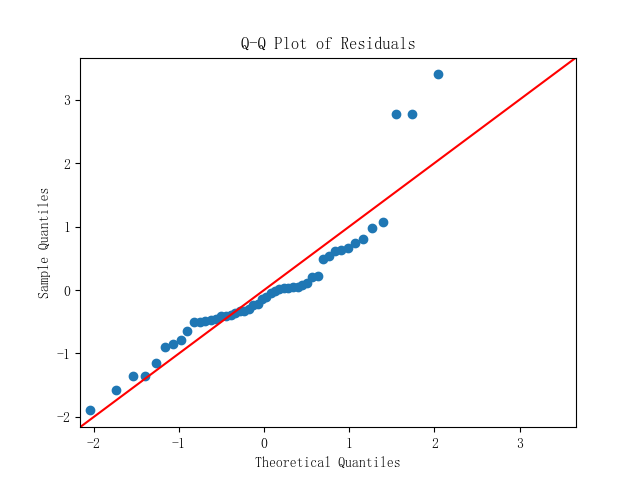

Supplement: Supplementary file 4 [file Image_4.png]
